# Coffee Consumption and Risk of Diabetic Angiopathy: Mediating Role of Gut Microbiota Revealed by Mendelian Randomization

**DOI:** 10.1002/fsn3.71728

**Published:** 2026-04-15

**Authors:** Wei Bao, Xiao‐Jia Huang, Ning Gu

**Affiliations:** ^1^ Nanjing Hospital of Chinese Medicine Affiliated to Nanjing University of Chinese Medicine Nanjing China

**Keywords:** coffee consumption, diabetic angiopathy, gut microbiota, Mendelian randomization

## Abstract

Diabetic angiopathy (DA) is a severe complication of diabetes mellitus, contributing significantly to morbidity and mortality. Although traditional mechanisms emphasize hyperglycemia‐induced oxidative stress and inflammation, emerging evidence highlights the role of gut microbiota in diabetes and its complications. Coffee consumption, rich in bioactive compounds, has been linked to metabolic health, but its relationship with DA remains unclear due to confounding factors. This study employed Mendelian randomization (MR) to investigate the causal effects of coffee consumption on DA and the mediating role of gut microbiota. Using genetic variants as instrumental variables, we analyzed summary data from large genome‐wide association studies on coffee consumption, gut microbiota, and DA. Results indicated a causal association between higher coffee consumption and increased DA risk, with specific gut microbial *Lawsonibacter sp002161175* partially mediating this relationship. Multivariable MR (MVMR) and Bayesian weighted MR (BWMR) supported the robustness of these findings. This study provides genetic evidence for a causal link between coffee intake and DA and suggests a potential mediating role of gut microbiota, offering insights for preventive strategies against diabetic vascular complications.

## Graphical abstract

This Mendelian randomization study reveals that coffee consumption causally increases the risk of diabetic angiopathy (DA). The gut microbiota, specifically *Lawsonibacter sp002161175*, partially mediates this relationship. These findings highlight the role of the gut microbiome in the diet–vascular disease axis and may inform future preventive strategies for diabetic vascular complications.

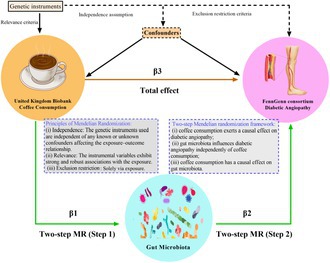

## 1 Introduction

Diabetic angiopathy (DA) is one of the most serious complications of diabetes mellitus, involving microvascular damage that manifests as cardiovascular disease, retinopathy, nephropathy, and neuropathy. DA significantly contributes to the increased morbidity and mortality in diabetic patients (Yun and Ko 2021). The pathogenesis of DA is primarily driven by chronic hyperglycemia‐induced oxidative stress and inflammatory responses, characterized by excessive reactive oxygen species generation and pro‐inflammatory cytokine release, which collectively promote vascular endothelial dysfunction and damage (Reynolds et al. 2023).

Recent research has expanded beyond these traditional mechanisms to emphasize the critical role of the gut microbiome in type 2 diabetes (T2DM) and its complications (Baars et al. 2024). The gut microbiota forms a complex ecosystem essential for maintaining metabolic and immune homeostasis. In diabetic individuals, gut dysbiosis, characterized by reduced microbial diversity, decreased abundance of beneficial taxa, expansion of pathobionts, and impaired intestinal barrier function, has been strongly associated not only with insulin resistance and dysglycemia but also with the development of both macro‐ and microangiopathy (Crudele et al. 2023). These findings suggest a pivotal interplay between gut microbial imbalance and diabetic vascular injury.

Coffee, one of the most widely consumed beverages globally, contains numerous bioactive compounds such as chlorogenic acid, caffeine, magnesium, potassium, and niacin. Accumulating observational evidence indicates that coffee intake may influence metabolic health (Barrea et al. 2023); however, its association with diabetic angiopathy remains inconsistent and inconclusive, likely due to residual confounding from lifestyle factors, physical activity levels, and reverse causality (Zhou et al. 2024). Notably, coffee consumption is known to modulate the composition and diversity of the gut microbiota (Saygili et al. 2024), raising the compelling hypothesis that gut microbes may mediate the relationship between coffee consumption and DA.

To overcome the limitations of conventional observational studies and strengthen causal inference, we applied Mendelian randomization, a genetic instrumental variable approach that uses genetic variants as proxies for modifiable exposures to estimate causal relationships while minimizing confounding and reverse causation (Sekula et al. 2016). Furthermore, we conducted the two‐step Mendelian randomization analyses to explore potential mediating pathways involving the gut microbiota (Carter et al. 2021). By integrating these analytical approaches, this study seeks to establish robust genetic evidence supporting a causal relationship between coffee consumption and DA, and to clarify the mediating role of the gut microbiota. These findings may contribute to the development of novel preventive and therapeutic interventions.

## 2 Materials and Methods

### 2.1 Study Design

**FIGURE 1:**
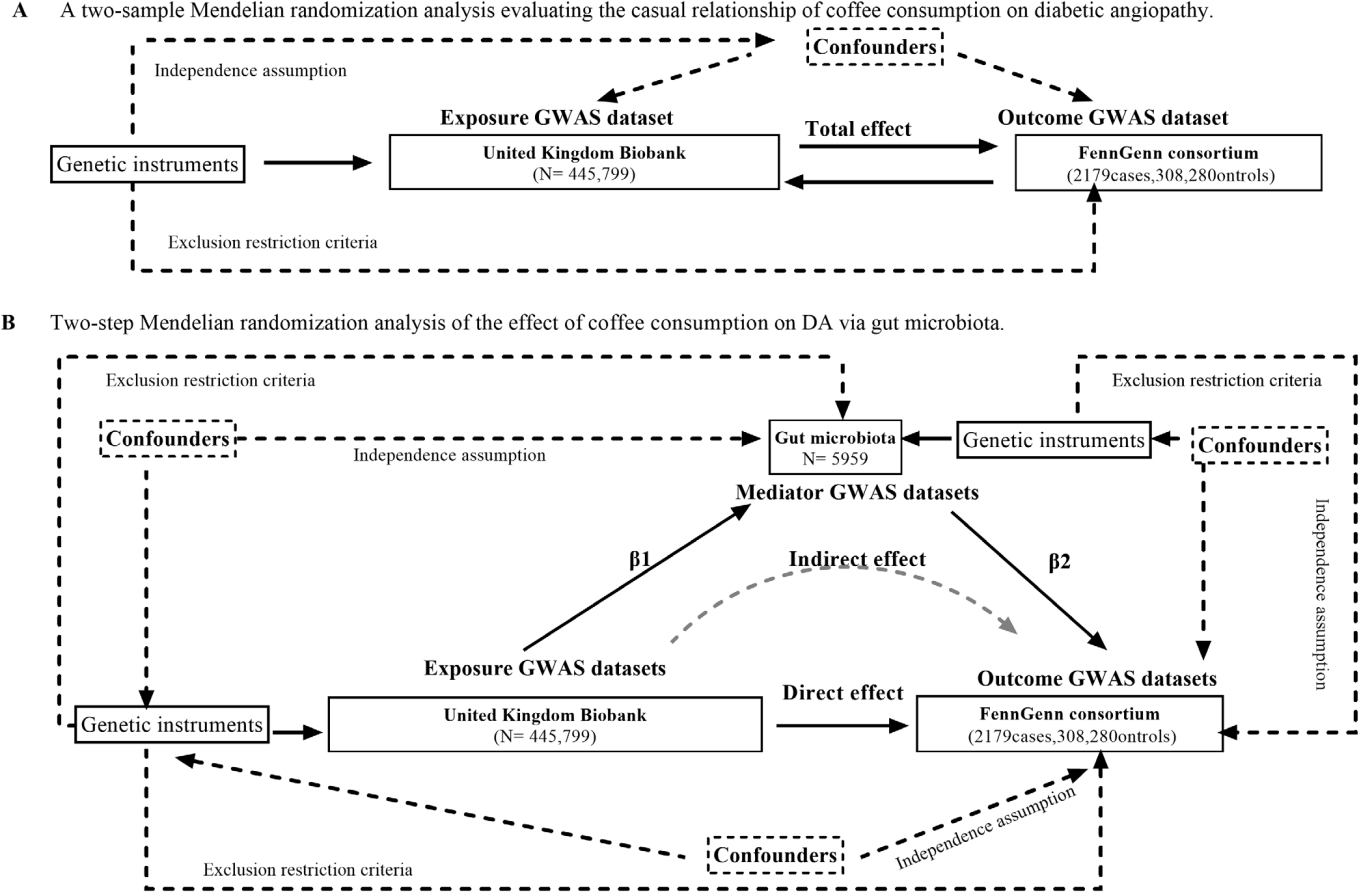
(A) Three core assumptions of Mendelian randomization. (B) Two‐step MR mediation framework for testing gut microbiota as a mediator of the coffee‐DA relationship.

This Mendelian randomization study was conducted and reported in accordance with the STROBE‐MR guidelines, with detailed items provided in File S1 (Skrivankova et al. 2021). The study design is illustrated in Figure 1, outlining the assumptions and framework of the two‐sample and mediation Mendelian randomization analyses. The validity of Mendelian randomization (MR) analysis relies on three core assumptions: (i) the selected genetic instruments must be robustly associated with the exposure of interest (relevance assumption); (ii) the instruments must not be confounded by common causes of the exposure and outcome (independence assumption); and (iii) the genetic variants must influence the outcome solely through the exposure, without operating through alternative pathways (exclusion restriction assumption). We first employed a bidirectional two‐sample MR approach to investigate the causal relationship between coffee consumption and diabetic angiopathy. We then conducted causal assessments of the gut microbiota, covering 473 microbial taxa (at various taxonomic levels), to identify specific bacterial genera that may serve as risk or protective factors in DA development. Finally, we employed a two‐step Mendelian randomization (MR) approach to examine the causal pathways involving coffee consumption as the exposure, gut microbiota as the mediator, and DA as the outcome. This analysis relied on three essential conditions: (i) a causal effect of the exposure on the outcome; (ii) an independent causal effect of the mediator on the outcome, conditional on the exposure; and (iii) a causal effect of the exposure on the mediator (Richmond and Davey Smith 2022).

### 2.2 Data Sources

Exposure data were extracted from GWAS summary statistics provided by Pirastu et al. (2022), comprising total coffee consumption (*N* = 105,037), decaffeinated coffee consumption (*N* = 62,072), ground coffee consumption (*N* = 72,276), instant coffee consumption (*N* = 180,764), and water consumption adjusted for coffee intake (*N* = 400,642). Coffee intake was assessed as cups/day and log_10_‐transformed (log_10_[cups/day + 1]); water intake was square‐root transformed. Participants were stratified by coffee type based on usual consumption, excluding those with unspecified type. Data for 473 gut microbial taxa were obtained from a genome‐wide association study catalog comprising 5959 individuals from the FINRISK 2002 cohort (Qin et al. 2022). The summary statistics for these taxa are publicly available in the GWAS Catalog (https://www.ebi.ac.uk/gwas/) under accession numbers GCST90032172 to GCST90032644. Genetic associations for diabetic angiopathy were sourced from the FinnGen consortium from the FinnGen R9 dataset (https://storage.googleapis.com/finngen‐public‐data‐r9/summary_stats/finngen_R9_DM_PERIPH_ANGIOPATHY.gz), which included 2179 cases and 308,280 controls. All GWAS data were derived from independent consortia to avoid sample overlap (Table 1). Ethical approval was not needed for this current study because it is a secondary analysis of previously published data.

**TABLE 1:** Comprehensive overview of GWAS datasets in this study.

Traits	Sample size	Consortium/authors	GWAS ID
Coffee consumption	105,037	UK biobank	ebi‐a‐GCST90096894
Decaffeinated coffee consumption	62,072	UK biobank	ebi‐a‐GCST90096908
Ground coffee consumption	72,276	UK biobank	ebi‐a‐GCST90096913
Instant coffee consumption	180,764	UK biobank	ebi‐a‐GCST90096914
Water consumption	400,642	UK biobank	ebi‐a‐GCST90096928
Gut microbiota	5959	Qin et al.	ebi‐a‐GCST90032172‐ebi‐a‐GCST90032644
Diabetic angiopathy	274,331	FinnGen	finngen_R9_DM_PERIPH_ANGIOPATHY

### 2.3 Selection of Instrumental Variables

First, instrumental variables (IVs) were selected based on a strong association with the exposure. Single‐nucleotide polymorphisms (SNPs) meeting the locus‐wide significance threshold (*p* < 5 × 10^−8^) were prioritized as IVs. In cases where insufficient genome‐wide significant SNPs were available, SNPs associated at *p* < 1 × 10^−5^ were included as alternatives, consistent with prior Mendelian randomization studies of the gut microbiome (Ma et al. 2024). Second, to ensure robustness of the IV–exposure association, variants with weak instrument statistics (*F*‐statistic < 10, calculated as beta^2^/SE^2^) were excluded (Table S1) (Burgess et al. 2011). Third, to avoid bias due to linkage disequilibrium (LD), we clumped SNPs based on an LD threshold of *R* ^2^ < 0.001 within a 10,000 kb window. To satisfy the assumptions that IVs are not supposed to be related to outcome or any confounding factors, after SNPs were all retrieved from GWAS summary data of outcome, every SNP was searched at the GWAS Catalog website (https://www.ebi.ac.uk/gwas) to examine for signs of pleiotropy and whether strongly associated confounding factors. Effect alleles were harmonized between exposure and outcome datasets, and palindromic (e.g., A/T or G/C) as well as ambiguous or duplicated SNPs were removed (Jia et al. 2025). Furthermore, independent sets of SNPs were used for the exposure and mediator to maintain genetic independence.

### 2.4 Mediation Effects of Gut Microbiota

A two‐step MR approach was used to evaluate the mediating role of gut microbiota in the relationship between coffee consumption and DA. Due to a strong negative phenotypic correlation between water and coffee consumption, coffee intake was included as a covariate in water consumption analyses. Multivariable MR (MVMR) was applied to assess the direct effect of coffee consumption on DA after adjusting for other risk factors. The proportion of mediation was calculated as (*β*₁ × *β*₂)/*β*₃ (Rogne et al. 2024), where *β*₁ represents the effect of coffee on gut microbiota, *β*₂ the effect of gut microbiota on DA, and *β*₃ the total effect of coffee on DA. Standard errors were estimated using bootstrap methods.

### 2.5 Statistical Analysis

Five MR methods were used to estimate causal effects, with inverse variance weighted (IVW) or Wald ratio models serving as the primary methods for causal relationship assessment. To further account for potential pleiotropy and weak instrument bias, we additionally applied Bayesian weighted Mendelian randomization (BWMR) as a sensitivity analysis. BWMR employs a Bayesian framework to detect outliers and provide more robust causal estimates by down‐weighting potentially invalid instruments. Heterogeneity was assessed using Cochran's *Q*, *I* ^2^, and *H* statistics, when heterogeneity was present, the results of random‐ effects IVW were considered reliable (Xiao et al. 2022). Considering multiple testing, the false discovery rate (FDR) based on Benjamini‐Hochberg approach was used for multiple testing correction. Causal evidence was considered significant when *p <* 0.05 & *p*_FDR_ < 0.05 and suggestive when *p* < 0.05 & *p*_FDR_ > 0.05. Sensitivity analyses included weighted median and MR‐Egger regression. Horizontal pleiotropy was evaluated via the intercept term in MR‐Egger regression; an intercept near zero (< 0.1) with *p* > 0.05 indicated no pleiotropy. The MR‐PRESSO method was used to detect outliers and recalculate estimates after their removal. All MR analyses and causal effect estimations were performed in R (version 4.5.1) using the TwoSampleMR package, whereas pleiotropy was assessed using MR‐ PRESSO. Bayesian Weighted Mendelian Randomization was implemented with the BWMR package. The code for the MR analysis is adapted from the following sources: https://github.com/JHZ752/Gut‐Microbiota_PBC and https://github.com/jiazhao97/BWMR.

## 3 Results

### 3.1 Bidirectional Two‐Sample Mendelian Randomization Analyses Between Coffee Consumption and DA

We obtained 2–43 SNPs associated with each of 5 consumption traits as IVs meeting universally accepted genome‐wide significance thresholds (*p* < 5 × 10^−8^, *r* ^2^ < 0.001, kb = 10,000). SNPs related to physical activity (rs66723169) (Narayan and Yoon 2022), cholesterol (rs1057868, rs11127048), BMI (rs4148155), and diabetes mellitus (rs7564708) were also removed for they were associated with the outcomes. Each IV was valid with an *F*‐statistic > 10, indicating a low risk of bias from weak instruments (Table S1).

We performed two‐sample MR analyses to investigate the causal relationships between four coffee consumption traits and diabetic angiopathy (DA). To enhance the robustness of our findings, we employed both the IVW method and the BWMR method, the latter of which accounts for potential weak instrument bias and pleiotropy.

Total coffee consumption showed a consistent and robust positive association with DA risk across both methods (IVW: OR = 2.211, 95% CI: 1.471–3.323, *p* = 0.0001, *p*_FDR_ = 0.007; BWMR: OR = 2.217, 95% CI: 1.467–3.349, *p* = 0.0001), providing strong evidence for a causal effect.

For the remaining coffee traits (ground, instant, and decaffeinated coffee consumption), the MR analyses were limited by a small number of genetic instruments (≤ 2 SNPs), leading to extremely wide confidence intervals, substantial heterogeneity, or conflicting results between IVW and BWMR. Due to these methodological concerns, we consider these findings unreliable and focus subsequent analyses on total coffee consumption. In contrast, water consumption was analyzed as a negative control. Despite evidence of significant heterogeneity and pleiotropy in sensitivity analyses (*p* < 0.05), the primary IVW and BWMR methods consistently showed no causal association with DA (IVW: OR = 1.15, 95% CI: 0.69–1.91, *p* = 0.587; BWMR: OR = 1.14, 95% CI: 0.67–1.92, *p* = 0.635), reinforcing the specificity of the coffee‐DA relationship (Table S2).

**FIGURE 2:**
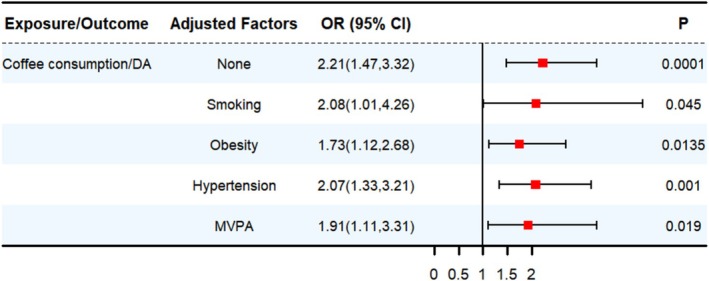
MVMR estimates derived from the IVW method to assess the causal effect of coffee consumption on DA. CI, confidence interval; OR, odds ratio.

We then conducted MVMR analysis to assess the direct effect of total coffee consumption on DA, after adjusting for potential confounders including smoking, obesity, hypertension, and moderate‐to‐vigorous physical activity (MVPA). The MVMR results were consistent with the univariable MR findings (Figure 2; Table S3), further supporting the robustness of the primary association.

Finally, reverse MR analysis showed no significant causal effect of DA on total coffee consumption (*p* > 0.05; Table S4), ruling out reverse causality.

### 3.2 Genetic Causal Effects of Gut Microbiota on DA

According to the IVW analysis, 28 causally linked gut microbiota were identified; however, these associations were no longer significant after FDR correction. Therefore, these findings were regarded as suggestive evidence of potential causal relationships and were subsequently subjected to further validation using BWMR. After excluding 21 microbiota that yielded inconsistent results in BWMR, a final total of 7 gut microbiota were found to have robust and consistent causal effects on DA.

**FIGURE 3:**
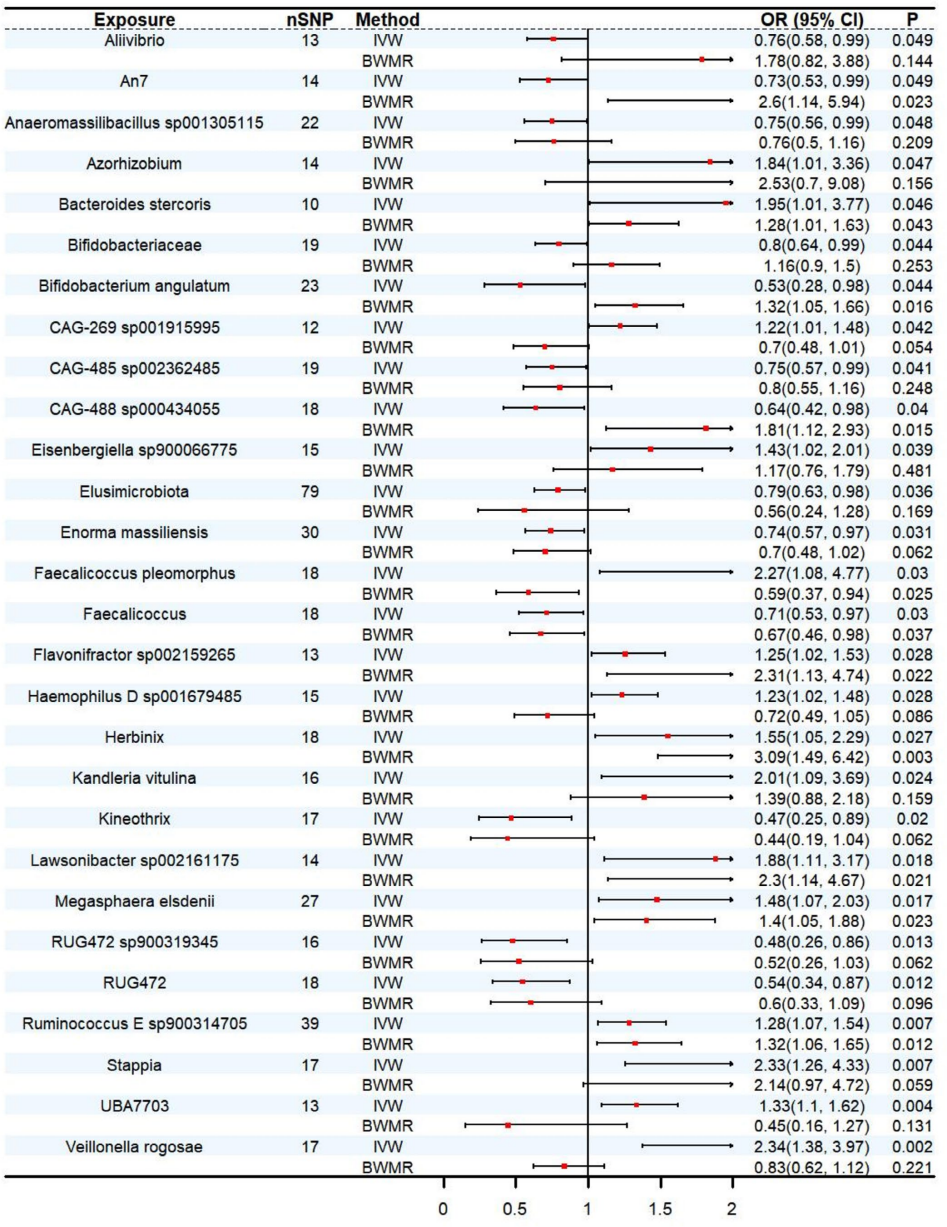
MR estimates of the causal effect of gut microbiota on DA. CI, confidence interval; OR, odds ratio.

*Faecalicoccus* (OR: 0.76, 95% CI: 0.578–0.999, *p* = 0.049) was identified as a potential protective factor. *Bacteroides stercoris* (OR: 1.254, 95% CI: 1.024–1.535, *p* = 0.028), *Flavonifractor sp002159265* (OR: 2.339, 95% CI: 1.377–3.973, *p* = 0.002), *Herbinix* (OR: 2.01, 95% CI: 1.094–3.693, *p* = 0.024), *Lawsonibacter sp002161175* (OR: 1.878, 95% CI: 1.113–3.169, *p* = 0.018), *Megasphaera elsdenii* (OR: 1.334, 95% CI: 1.096–1.622, *p* = 0.004), and *Ruminococcus E sp900314705* (OR: 1.283, 95% CI: 1.069–1.539, *p* = 0.007) abundance in stool are risk factors for DA; their elevated abundance increases the risk of DA (Figure 3; Tables S5 and S6).

### 3.3 Genetic Causal Effects Between Coffee Consumption and Gut Microbiota

Our initial findings indicated a causal relationship between coffee consumption, gut microbiota and DA. To gain deeper insights into the underlying mechanisms, we conducted a two‐step MR analysis to assess potential mediating effects. The analysis showed that total coffee consumption was positively associated with the abundance of *Lawsonibacter sp002161175* (OR: 1.106; 95% CI: 1.002–1.222; *p* = 0.044) in stool (Table S7).

### 3.4 Mediation Analysis

**FIGURE 4:**
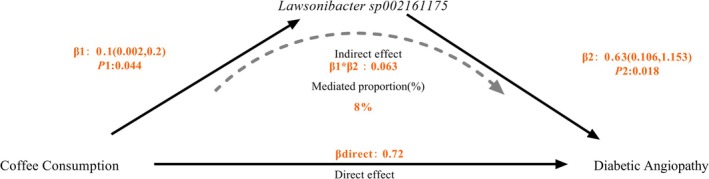
Schematic diagram of the mediation effect by *Lawsonibacter* sp0*02161175*.

Building upon the previous analysis, we identified a key gut microbiota mediating the causal link between total coffee consumption and DA; the results indicated a partial mediation effect, but it was not statistically significant. The *Lawsonibacter sp002161175* abundance in stool accounted for 8% of the total effect between coffee consumption and DA (Figure 4).

### 3.5 Sensitivity Analysis

**FIGURE 5:**
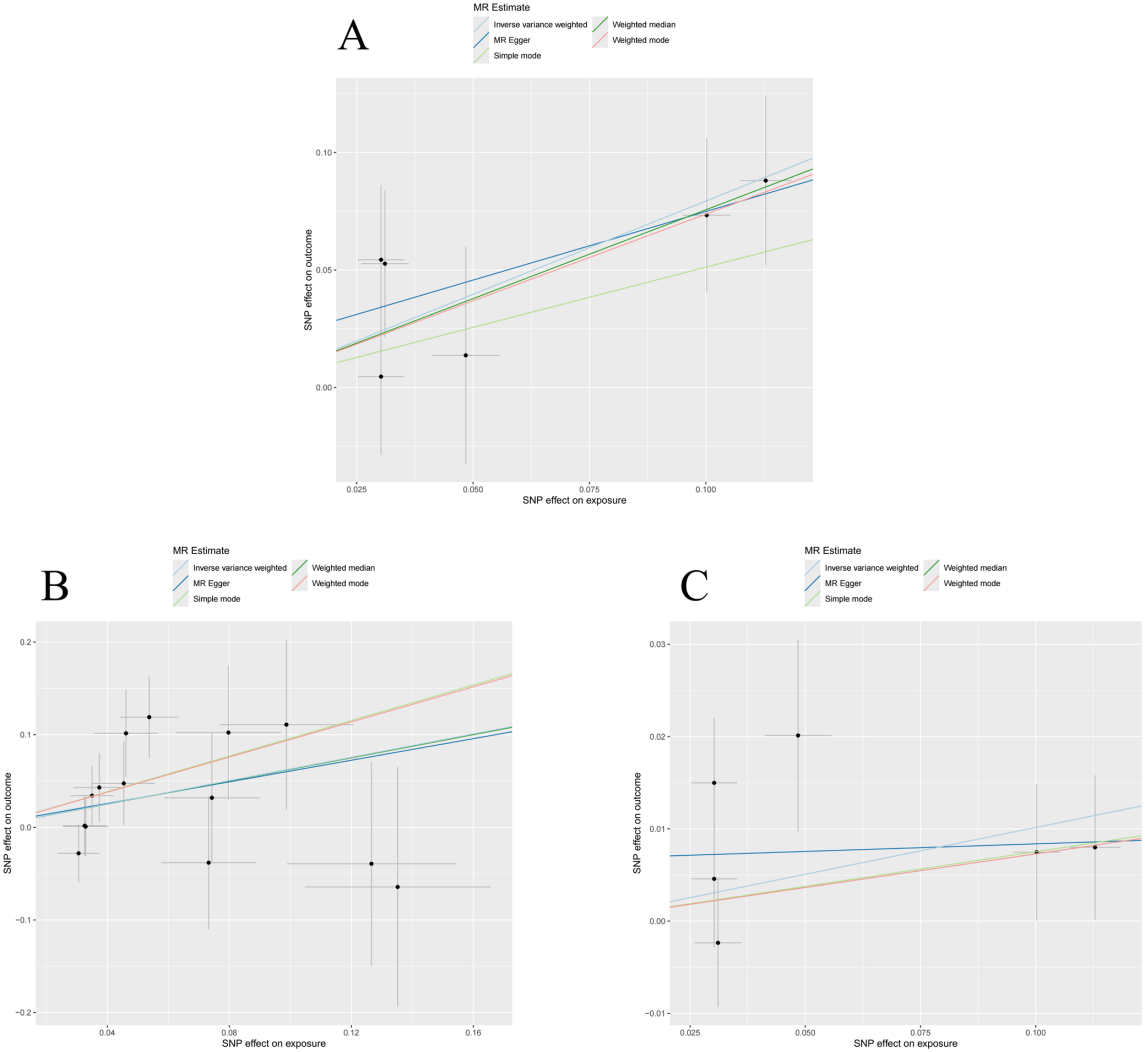
Scatter plots of SNP effect estimates for (A) coffee consumption on diabetic angiopathy (DA), (B) *Lawsonibacter* sp0*02161175 abundance* on DA, and (C) coffee consumption on *Lawsonibacter* sp0*02161175* abundance. Each point represents a single nucleotide polymorphism (SNP), with horizontal and vertical lines indicating 95% confidence intervals. The slopes of the lines correspond to the causal estimates derived from the inverse‐variance weighted (IVW) method.

To assess the robustness of the causal estimates, we performed a series of sensitivity analyses. The heterogeneity test showed no significant heterogeneity according to Cochran's *Q* statistic (*p* > 0.05), indicating consistent effect estimates across genetic variants. The MR‐Egger regression intercept was close to zero (*p* > 0.05), and the MR‐PRESSO global test did not identify any significant outliers (*p* > 0.05), suggesting the absence of directional horizontal pleiotropy. The scatter plot (Figure 5) visually demonstrates that the SNP effect estimates closely align with the IVW regression line, and the funnel plot (Figure S1) is largely symmetric, further excluding potential bias. In the leave‐one‐out analysis, the pooled effect estimates from the remaining SNPs after sequentially removing each SNP were consistently in the same direction as the original point estimate (Figure S2). Collectively, these analyses substantiate the robustness of the observed causal association between coffee consumption and diabetic angiopathy, indicating that the causal relationships of the positive findings were highly robust.

## 4 Discussion

Utilizing large‐scale genetic association data within a Mendelian randomization framework, this study provides evidence supporting a potential causal effect of coffee consumption on increased risk of diabetic angiopathy. After adjusting for lifestyle and metabolic confounders, MVMR analyses further confirmed that total coffee consumption independently influences the risk of diabetic angiopathy. Moreover, through a two‐step MR approach, we identified *Lawsonibacter sp002161175* as potential mediators in the pathway linking coffee consumption to DA.

The health effects of coffee on type 2 diabetes (T2D), cardiovascular disease (CVD), and all‐cause mortality remain controversial. Several prospective studies have suggested that moderate coffee intake is associated with reduced risk of cardiometabolic diseases (Lu et al. 2025). Among never‐smoking individuals with T2D, moderate consumption, typically defined as 2 to 4 cups per day, was linked to lower incidence of CVD and chronic kidney disease, with no adverse effects observed even at higher intake levels (Liu et al. 2023). A cross‐sectional study also reported that caffeine intake was associated with reduced risk of diabetic retinopathy in T2D patients without other late complications, though these findings lack support from experimental models (Alcubierre et al. 2023). In contrast, other prospective cohorts found no significant association between coffee consumption and risk of microvascular complications in T2D patients (Lin et al. 2025). A cross‐sectional study indicated that high coffee consumption was associated with central obesity and noted differential effects between ground and instant coffee on metabolic syndrome (Wong et al. 2024). A previous MR study also provided limited evidence for causal effects of coffee consumption on CVD risk (Yuan et al. 2021). These inconsistencies highlight fundamental limitations of observational epidemiology, particularly residual confounding. Coffee consumers may exhibit different lifestyle patterns such as physical activity level and smoking status that are not fully accounted for in statistical adjustments (Etminan and Rezaeianzadeh 2023). Our multivariable MR analysis, which adjusted for several lifestyle factors, attenuated the coffee‐DA association, supporting the role of confounding. However, the persistence of direct effects in some models warrants further investigation.

We identified seven gut microbial taxa with robust causal links to DA after rigorous validation using BWMR. Among these, *Faecalicoccus* (OR: 0.76, 95% CI: 0.578–0.999, *p* = 0.049) was identified as a potential protective factor, consistent with previous studies associating this genus with improved metabolic health (Liu et al. 2021). Conversely, risk taxa including *Bacteroides stercoris* (Ni et al. 2023) and *Ruminococcus E sp900314705* (Clark and Mach 2016) have been linked to pro‐inflammatory states and intestinal barrier dysfunction in other studies. *Megasphaera elsdenii* (He et al. 2022) was found to be significantly enriched in patients with diabetic kidney disease, suggesting its potential involvement in the pathogenesis of diabetic vascular complications through its association with gut microbiota dysbiosis. *Flavonifractor sp002159265* (Cui et al. 2022), a species within the genus *Flavonifractor*, is associated with reduced insulin sensitivity and may contribute to diabetic vascular disease by promoting insulin resistance and systemic inflammation. Our findings provide genetic evidence substantiating the role of gut microbiota in modulating diabetic vascular complications, potentially through mechanisms involving systemic inflammation and immune activation. Our findings genetically substantiate growing evidence that the gut microbiome modulates diabetic vascular complications, potentially through mechanisms involving systemic inflammation and immune activation.

Although the relationship between coffee consumption and gut microbiota is well‐established, our study novelly examined the microbial mediation effect between coffee intake and DA. Our findings are consistent with previous research indicating that coffee consumption causally increases the abundance of *Lawsonibacter sp002161175* (Manghi et al. 2024), previously reported to be associated with the development of abdominal aortic aneurysm (Lyu et al. 2025).

The observed positive association between coffee and DA may be biologically plausible. Although coffee contains antioxidants, it is also a source of caffeine and cafestol, which have been linked to increased hypertension risk (Zhang et al. 2011) and elevated serum cholesterol in susceptible individuals (Olthof et al. 2001). Both hypertension and hypercholesterolemia are established risk factors for microvascular damage. Furthermore, coffee compounds may directly interact with gut epithelium and microbiota, influencing both innate and adaptive immune responses in the gut and affect inflammatory bowel disease (Saygili et al. 2024).

This study has several strengths. First, we combined BWMR and MVMR to identify the most relevant coffee consumption traits among correlated phenotypes. Second, species‐level analysis of gut microbiota provided more precise characterization of mediating taxa. Third, adjustment for lifestyle factors enhanced result reliability.

However, several limitations must be acknowledged. First, primarily European ancestry data limit generalizability to other populations. Second, insufficient data precluded quantitative analysis of coffee‐DA dose response. Third, a key limitation is that after applying a stringent false discovery rate correction (*p*_FDR_ < 0.05), none of the initially identified gut microbial taxa retained statistical significance in their association with DA. This attenuation reflects the inherent trade‐off between multiple testing control and statistical power in high‐dimensional omics data, particularly given the modest sample size of the gut microbiota GWAS. Consequently, our findings regarding microbial mediation should be interpreted as exploratory and hypothesis‐generating, warranting validation in larger, well‐powered cohorts.

## 5 Conclusions

In conclusion, our MR study provides genetic evidence supporting a causal effect of coffee consumption on diabetic angiopathy risk and identifies specific gut microbial taxa implicated in DA pathogenesis. Although suggestive evidence indicates gut microbiota may mediate coffee's effects, mediation was not statistically significant. Future research should prioritize larger GWAS of gut microbiome to enhance mediation analysis power, investigate specific bioactive components in coffee, and explore these relationships in diverse populations. Our findings contribute to a more nuanced understanding of the diet‐microbiome‐disease axis in diabetes complications.

## Author Contributions

**Xiao‐Jia Huang:** validation, visualization, project administration, writing – original draft. **Wei Bao:** investigation, conceptualization, data curation, software, formal analysis, methodology. **Ning Gu:** writing – review and editing, supervision, funding acquisition.

## Funding

The authors have nothing to report.

## Conflicts of Interest

The authors declare no conflicts of interest.

## Supporting information


**File S1:** STROBE—MR checklist.

**Table S1:** Instrumental variable characteristics and *F*‐statistics.
**Table S2:** Sensitivity analyses for coffee consumption and DA.
**Table S3:** Multivariable MR results adjusting for confounders.
**Table S4:** Reverse MR results (DA on coffee consumption).
**Table S5:** Full list of gut microbiota—DA causal estimates.
**Table S6:** BWMR validation results for gut microbiota.
**Table S7:** Causal effects of coffee consumption on gut microbiota.

**Figure S1:** Funnel plot of the causal effect for (A) coffee consumption on diabetic angiopathy (DA), (B) *Lawsonibacter sp002161175 abundance* on DA, and (C) coffee consumption on *Lawsonibacter sp002161175* abundance.
**Figure S2:** MR leave‐one out sensitivity analysis for (A) coffee consumption on diabetic angiopathy (DA), (B) *Lawsonibacter sp002161175 abundance* on DA, and (C) coffee consumption on *Lawsonibacter sp002161175* abundance.

## Data Availability

All GWAS summary data sources have been cited within the manuscript. The code used for the analyses is available at https://github.com/differentbao/coffee‐gut‐microbiota‐da.
