# Supplementary material for: Coffee Consumption and Risk of Diabetic Angiopathy: Mediating Role of Gut Microbiota Revealed by Mendelian Randomization
Source: Food Sci Nutr. 2026 Apr 15;14(4):e71728. doi: 10.1002/fsn3.71728 (PMC13083039; doi:10.1002/fsn3.71728)
Supplement: Supplementary file 1 — File S1: STROBE—MR checklist. [file FSN3-14-e71728-s002.docx]

**STROBE-MR checklist of recommended items to address in reports of Mendelian randomization studies**^1^ ^2^

| **Item No.** | **Section** | **Checklist item** | **Page No.** | **Relevant text from manuscript** |
| --- | --- | --- | --- | --- |
| 1 | **TITLE and ABSTRACT** | Indicate Mendelian randomization (MR) as the study’s design in the title and/or the abstract if that is a main purpose of the study | 1 | Title: "Coffee Consumption and Risk of Diabetic Angiopathy: Mediating Role of Gut Microbiota Revealed by Mendelian Randomization" Abstract: "This study employed Mendelian randomization (MR) to investigate the causal effects of coffee consumption on DA and the mediating role of gut microbiota...Multivariable MR (MVMR) and Bayesian weighted MR (BWMR) supported the robustness of these findings." |
|  | **INTRODUCTION** |  |  |  |
| 2 | **Background** | Explain the scientific background and rationale for the reported study. What is the exposure? Is a potential causal relationship between exposure and outcome plausible? Justify why MR is a helpful method to address the study question | 1-2 | "Coffee consumption, rich in bioactive compounds, has been linked to metabolic health, but its relationship with DA remains unclear due to confounding factors...To overcome the limitations of conventional observational studies and strengthen causal inference, we applied Mendelian randomization, a genetic instrumental variable approach...while minimizing confounding and reverse causation." |
| 3 | **Objectives** | State specific objectives clearly, including pre-specified causal hypotheses (if any). State that MR is a method that, under specific assumptions, intends to estimate causal effects | 2 | "By integrating these analytical approaches, this study seeks to establish robust genetic evidence supporting a causal relationship between coffee consumption and DA, and to clarify the mediating role of the gut microbiota. These findings may contribute to the development of novel preventive and therapeutic interventions." |
|  | **METHODS** |  |  |  |
| 4 | **Study design and data sources** | Present key elements of the study design early in the article. Consider including a table listing sources of data for all phases of the study. For each data source contributing to the analysis, describe the following: | 2-3 | Study Design: "This Mendelian randomization study was conducted and reported in accordance with the STROBE-MR guidelines. The study design is illustrated in Figure 1..." Data Sources: "Exposure data were obtained from GWAS summary statistics by Pirastu et al. (2022)...Data for 473 gut microbial taxa were obtained from...the FINRISK 2002 cohort...Genetic associations for diabetic angiopathy were sourced from the FinnGen consortium..." |
|  | a) | Setting: Describe the study design and the underlying population, if possible. Describe the setting, locations, and relevant dates, including periods of recruitment, exposure, follow-up, and data collection, when available. | 3 | Data Sources: "Exposure data were obtained from GWAS summary statistics by Pirastu et al. (2022), comprising...UK biobank...Data for 473 gut microbial taxa were obtained from...the FINRISK 2002 cohort...Genetic associations for diabetic angiopathy were sourced from the FinnGen consortium from the FinnGen R9 dataset." (All derived from European populations). |
|  | b) | Participants: Give the eligibility criteria, and the sources and methods of selection of participants. Report the sample size, and whether any power or sample size calculations were carried out prior to the main analysis | 3 | Data Sources: "Exposure data were obtained from GWAS summary statistics by Pirastu et al. (2022), comprising total coffee consumption(N=105,037)...Data for 473 gut microbial taxa were obtained from a genome-wide association study catalog comprising 5,959 individuals...Genetic associations for diabetic angiopathy were sourced from the FinnGen consortium...which included 2,179 cases and 308,280 controls." |
|  | c) | Describe measurement, quality control and selection of genetic variants | 3-4 | Selection of Instrumental Variables: "Firstly, instrumental variables (IVs) were selected based on a strong association with the exposure. Single-nucleotide polymorphisms (SNPs) meeting the locus-wide significance threshold (*P*< 5×10⁻⁸) were prioritized...we clumped SNPs based on an LD threshold of R² < 0.001 within a 10,000 kb window...Effect alleles were harmonized...and palindromic...SNPs were removed." |
|  | d) | For each exposure, outcome, and other relevant variables, describe methods of assessment and diagnostic criteria for diseases | 3 | Data Sources: "Coffee intake was assessed as cups/day and log10‑transformed...Data for 473 gut microbial taxa were obtained from a genome-wide association study...Genetic associations for diabetic angiopathy were sourced from the FinnGen consortium..." (Specific diagnostic criteria for DA are inherent to the FinnGen dataset). |
|  | e) | Provide details of ethics committee approval and participant informed consent, if relevant | 3 | Data Sources: "Ethical approval was not needed for this current study because it is a secondary analysis of previously published data." (Original GWAS studies would have obtained ethical approval). |
| 5 | **Assumptions** | Explicitly state the three core IV assumptions for the main analysis (relevance, independence and exclusion restriction) as well assumptions for any additional or sensitivity analysis | 2 | Study Design: "The validity of Mendelian randomization (MR) analysis relies on three core assumptions: (i) the selected genetic instruments must be robustly associated with the exposure of interest (relevance assumption); (ii) the instruments must not be confounded by common causes of the exposure and outcome (independence assumption); and (iii) the genetic variants must influence the outcome solely through the exposure, without operating through alternative pathways (exclusion restriction assumption)." |
| 6 | **Statistical methods: main analysis** | Describe statistical methods and statistics used | 4-5 |  |
|  | a) | Describe how quantitative variables were handled in the analyses (i.e., scale, units, model) | 3,5 | Data Sources: "Coffee intake was assessed as cups/day and log10‑transformed..." Statistical Analysis: "Five MR methods were used to estimate causal effects, with inverse variance weighted (IVW) or Wald ratio models serving as the primary methods...Bayesian weighted Mendelian randomization (BWMR) as a sensitivity analysis." |
|  | b) | Describe how genetic variants were handled in the analyses and, if applicable, how their weights were selected | 3-4 | Selection of Instrumental Variables: "Firstly, instrumental variables (IVs) were selected based on a strong association with the exposure...we clumped SNPs...Effect alleles were harmonized between exposure and outcome datasets... independent sets of SNPs were used for the exposure and mediator." |
|  | c) | Describe the MR estimator (e.g. two-stage least squares, Wald ratio) and related statistics. Detail the included covariates and, in case of two-sample MR, whether the same covariate set was used for adjustment in the two samples | 4-5 | Statistical Analysis: "Five MR methods were used to estimate causal effects, with inverse variance weighted (IVW) or Wald ratio models serving as the primary methods...we additionally applied Bayesian weighted Mendelian randomization (BWMR) as a sensitivity analysis...Multivariable MR (MVMR) was applied to assess the direct effect of coffee consumption on DA after adjusting for other risk factors." (As this is two-sample MR using summary statistics, covariate adjustment is at the GWAS level and assumed to be consistent across samples). |
|  | d) | Explain how missing data were addressed | N/A | N/A |
|  | e) | If applicable, indicate how multiple testing was addressed | 4-5 | Statistical Analysis: "Considering multiple testing, the false discovery rate (FDR) based on Benjamini-Hochberg approach was used for multiple testing correction. Causal evidence was considered significant when *P*<0.05&*P*_FDR_ < 0.05 and suggestive when *P*<0.05&*P*_FDR_>0.05." |
| 7 | **Assessment of assumptions** | Describe any methods or prior knowledge used to assess the assumptions or justify their validity | 4-5 | Selection of IVs: "variants with weak instrument statistics (F-statistic < 10...were excluded" (Relevance). Statistical Analysis: "Sensitivity analyses included weighted median and MR-Egger regression. Horizontal pleiotropy was evaluated via the intercept term in MR-Egger regression...The MR-PRESSO method was used to detect outliers..." (Independence/Exclusion restriction). |
| 8 | **Sensitivity analyses and additional analyses** | Describe any sensitivity analyses or additional analyses performed (e.g. comparison of effect estimates from different approaches, independent replication, bias analytic techniques, validation of instruments, simulations) | 5 | Statistical Analysis: "Sensitivity analyses included weighted median and MR-Egger regression...The MR-PRESSO method was used to detect outliers and recalculate estimates after their removal...Bayesian weighted Mendelian randomization (BWMR) as a sensitivity analysis...Multivariable MR (MVMR)...bidirectional two-sample MR approach...two-step MR approach." |
| 9 | **Software and pre-registration** |  |  |  |
|  | a) | Name statistical software and package(s), including version and settings used | 5 | Statistical Analysis: "All MR analyses and causal effect estimations were performed in R (version 4.5.1) using the TwoSampleMR package, while pleiotropy was assessed using MR- PRESSO. Bayesian Weighted Mendelian Randomization was implemented with the BWMR package." |
|  | b) | State whether the study protocol and details were pre-registered (as well as when and where) | N/A | N/A |
|  | **RESULTS** |  |  |  |
| 10 | **Descriptive data** |  |  |  |
|  | a) | Report the numbers of individuals at each stage of included studies and reasons for exclusion. Consider use of a flow diagram | 3,5 | Data Sources: Sample sizes for each GWAS are reported in Table 1. Results: "We obtained 2 to 43 SNPs associated with each of 5 consumption traits as IVs...SNPs related to physical activity...cholesterol...BMI...and diabetes mellitus...were also removed..." (Further details on SNP exclusion are implied but not in a flow diagram). |
|  | b) | Report summary statistics for phenotypic exposure(s), outcome(s), and other relevant variables (e.g. means, SDs, proportions) | 3 | Summary statistics are inherent to the GWAS datasets and not presented in this paper, as it is a secondary analysis. |
|  | c) | If the data sources include meta-analyses of previous studies, provide the assessments of heterogeneity across these studies | N/A | The GWAS data sources are from single consortia, not meta-analyses of multiple studies. |
|  | d) | For two-sample MR:  i.  Provide justification of the similarity of the genetic variant-exposure associations between the exposure and outcome samples  ii.  Provide information on the number of individuals who overlap between the exposure and outcome studies | 3 | Data Sources: "All GWAS data were derived from independent consortia to avoid sample overlap." (The UK Biobank, FINRISK, and FinnGen are distinct cohorts, minimizing bias from sample overlap). |
| 11 | **Main results** |  |  |  |
|  | a) | Report the associations between genetic variant and exposure, and between genetic variant and outcome, preferably on an interpretable scale | 5, S1 | Results: "We obtained 2 to 43 SNPs associated with each of 5 consumptiosn traits as IVs..." (Full SNP lists and their associations are provided in supplementary tables, e.g., Table S1). |
|  | b) | Report MR estimates of the relationship between exposure and outcome, and the measures of uncertainty from the MR analysis, on an interpretable scale, such as odds ratio or relative risk per SD difference | 5-6 | Results: "Total coffee consumption showed a consistent and robust positive association with DA risk across both methods (IVW: OR = 2.211, 95% CI: 1.471--3.323, P = 0.0001,*P*_FDR_ = 0.007; BWMR: OR = 2.217, 95% CI: 1.467--3.349, *P*= 0.0001)..." |
|  | c) | If relevant, consider translating estimates of relative risk into absolute risk for a meaningful time period | N/A | Not performed. |
|  | d) | Consider plots to visualize results (e.g. forest plot, scatterplot of associations between genetic variants and outcome versus between genetic variants and exposure) | 6-8 | Results: The results are visualized in Figure 2 (MVMR estimates), Figure 3 (gut microbiota on DA), Figure 4 (mediation diagram), and Figure 5 (scatter plots). |
| 12 | **Assessment of assumptions** |  |  |  |
|  | a) | Report the assessment of the validity of the assumptions | 8 | Sensitivity Analysis: "The MR-Egger regression intercept was close to zero (*P* > 0.05), and the MR-PRESSO global test did not identify any significant outliers (*P* > 0.05), suggesting the absence of directional horizontal pleiotropy." |
|  | b) | Report any additional statistics (e.g., assessments of heterogeneity across genetic variants, such as *I^2^*, Q statistic or E-value) | 8 | Sensitivity Analysis: "The heterogeneity test showed no significant heterogeneity according to Cochran's Q statistic (*P* > 0.05), indicating consistent effect estimates across genetic variants." (I² and H statistics are mentioned in Methods but results not explicitly stated in main text, likely in supplement). |
| 13 | **Sensitivity analyses and additional analyses** |  |  |  |
|  | a) | Report any sensitivity analyses to assess the robustness of the main results to violations of the assumptions | 8 | Sensitivity Analysis: Describes the results of MR-Egger, MR-PRESSO, Cochran's Q, leave-one-out analysis, and funnel plot, all supporting the robustness of the findings. |
|  | b) | Report results from other sensitivity analyses or additional analyses | 5-8 | Results: Reports on BWMR, MVMR (Figure 2), and reverse MR analysis. |
|  | c) | Report any assessment of direction of causal relationship (e.g., bidirectional MR) | 6 | Results: "Finally, reverse MR analysis showed no significant causal effect of DA on total coffee consumption (*P* > 0.05; Table S4), ruling out reverse causality." |
|  | d) | When relevant, report and compare with estimates from non-MR analyses | 9 | Discussion: "A previous MR study also provided limited evidence for causal effects of coffee consumption on CVD risk...These inconsistencies highlight fundamental limitations of observational epidemiology, particularly residual confounding." |
|  | e) | Consider additional plots to visualize results (e.g., leave-one-out analyses) | 8 | Sensitivity Analysis: "...the funnel plot (Figure S1) is largely symmetric...In the leave-one-out analysis, the pooled effect estimates...were consistently in the same direction..." (These are supplementary figures). |
|  | **DISCUSSION** |  |  |  |
| 14 | **Key results** | Summarize key results with reference to study objectives | 9 | Discussion: "Utilizing large-scale genetic association data within a Mendelian randomization framework, this study provides evidence supporting a potential causal effect of coffee consumption on increased risk of diabetic angiopathy...we identified *Lawsonibacter sp002161175* as potential mediators in the pathway linking coffee consumption to DA." |
| 15 | **Limitations** | Discuss limitations of the study, taking into account the validity of the IV assumptions, other sources of potential bias, and imprecision. Discuss both direction and magnitude of any potential bias and any efforts to address them | 10 | Discussion: "However, several limitations must be acknowledged. First, primarily European ancestry data limit generalizability...Second, insufficient data precluded quantitative analysis of coffee-DA dose response. Third...after applying a stringent false discovery rate correction...none of the initially identified gut microbial taxa retained statistical significance...this attenuation reflects the inherent trade-off between multiple testing control and statistical power..." |
| 16 | **Interpretation** |  |  |  |
|  | a) | Meaning: Give a cautious overall interpretation of results in the context of their limitations and in comparison with other studies | 9-10 | Discussion: The authors compare their findings with previous observational and MR studies, noting inconsistencies and discussing potential reasons such as residual confounding. They interpret their findings cautiously, especially regarding the mediation analysis. |
|  | b) | Mechanism: Discuss underlying biological mechanisms that could drive a potential causal relationship between the investigated exposure and the outcome, and whether the gene-environment equivalence assumption is reasonable. Use causal language carefully, clarifying that IV estimates may provide causal effects only under certain assumptions | 10 | Discussion: "The observed positive association between coffee and DA may be biologically plausible...coffee is also a source of caffeine and cafestol, which have been linked to increased hypertension risk...and elevated serum cholesterol...Furthermore, coffee compounds may directly interact with gut epithelium and microbiota..." |
|  | c) | Clinical relevance: Discuss whether the results have clinical or public policy relevance, and to what extent they inform effect sizes of possible interventions | 10,11 | Discussion: "Our findings contribute to a more nuanced understanding of the diet-microbiome-disease axis in diabetes complications." Conclusions: "...offering insights for preventive strategies against diabetic vascular complications." |
| 17 | **Generalizability** | Discuss the generalizability of the study results (a) to other populations, (b) across other exposure periods/timings, and (c) across other levels of exposure | 10 | Discussion: "First, primarily European ancestry data limit generalizability to other populations. Second, insufficient data precluded quantitative analysis of coffee-DA dose response." |
|  | **OTHER INFORMATION** |  |  |  |
| 18 | **Funding** | Describe sources of funding and the role of funders in the present study and, if applicable, sources of funding for the databases and original study or studies on which the present study is based | 11 | Funding: "Not applicable." |
| 19 | **Data and data sharing** | Provide the data used to perform all analyses or report where and how the data can be accessed, and reference these sources in the article. Provide the statistical code needed to reproduce the results in the article, or report whether the code is publicly accessible and if so, where | 3,11 | Data Availability Statement: "All GWAS summary data sources have been cited within the manuscript...." |
| 20 | **Conflicts of Interest** | All authors should declare all potential conflicts of interest | 11 | Conflicts of Interest: "The authors declare no conflicts of interest." |

This checklist is copyrighted by the Equator Network under the Creative Commons Attribution 3.0 Unported (CC BY 3.0) license.

1. Skrivankova VW, Richmond RC, Woolf BAR, Yarmolinsky J, Davies NM, Swanson SA, et al. Strengthening the Reporting of Observational Studies in Epidemiology using Mendelian Randomization (STROBE-MR) Statement. JAMA. 2021;under review.

2. Skrivankova VW, Richmond RC, Woolf BAR, Davies NM, Swanson SA, VanderWeele TJ, et al. Strengthening the Reporting of Observational Studies in Epidemiology using Mendelian Randomisation (STROBE-MR): Explanation and Elaboration. BMJ. 2021;375:n2233.
