# Supplementary material for: Coffee Consumption and Risk of Diabetic Angiopathy: Mediating Role of Gut Microbiota Revealed by Mendelian Randomization
Source: Food Sci Nutr. 2026 Apr 15;14(4):e71728. doi: 10.1002/fsn3.71728 (PMC13083039; doi:10.1002/fsn3.71728)
Supplement: Supplementary file 3 — Figure S1: Funnel plot of the causal effect for (A) coffee consumption on diabetic angiopathy (DA), (B) Lawsonibacter sp002161175 abundance on DA, and (C) coffee consumption on Lawsonibacter sp002161175 abundance. Figure S2: MR leave‐one out sensitivity analysis for (A) coffee consumption on diabetic angiopathy (DA), (B) Lawsonibacter sp002161175 abundance on DA, and (C) coffee consumption on Lawsonibacter sp002161175 abundance. [file FSN3-14-e71728-s003.docx]

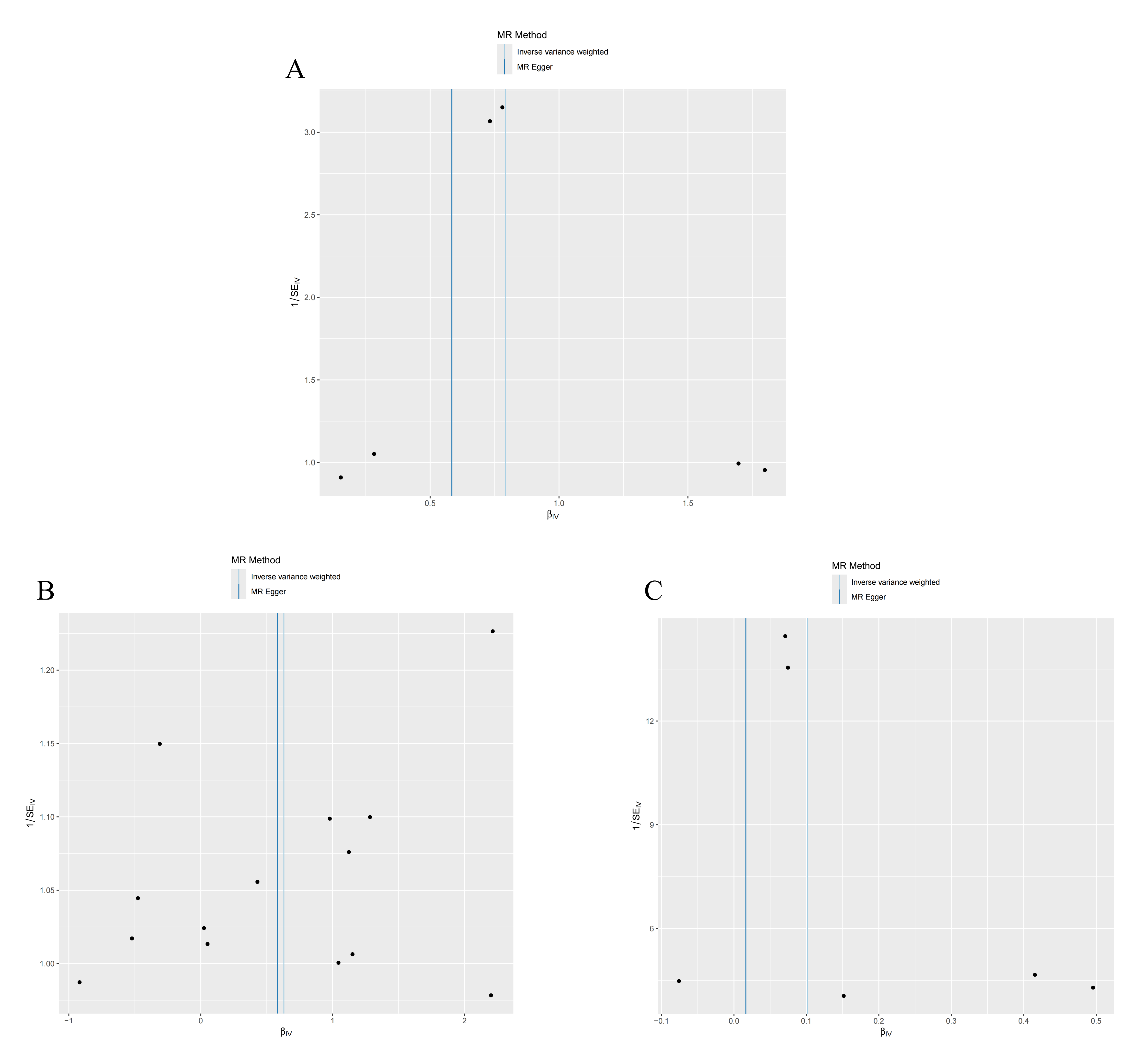


Figure S1. Funnel plot of the causal effect for (A) coffee consumption on diabetic angiopathy (DA), (B) *Lawsonibacter sp002161175 abundance* on DA, and (C) coffee consumption on *Lawsonibacter sp002161175* abundance.


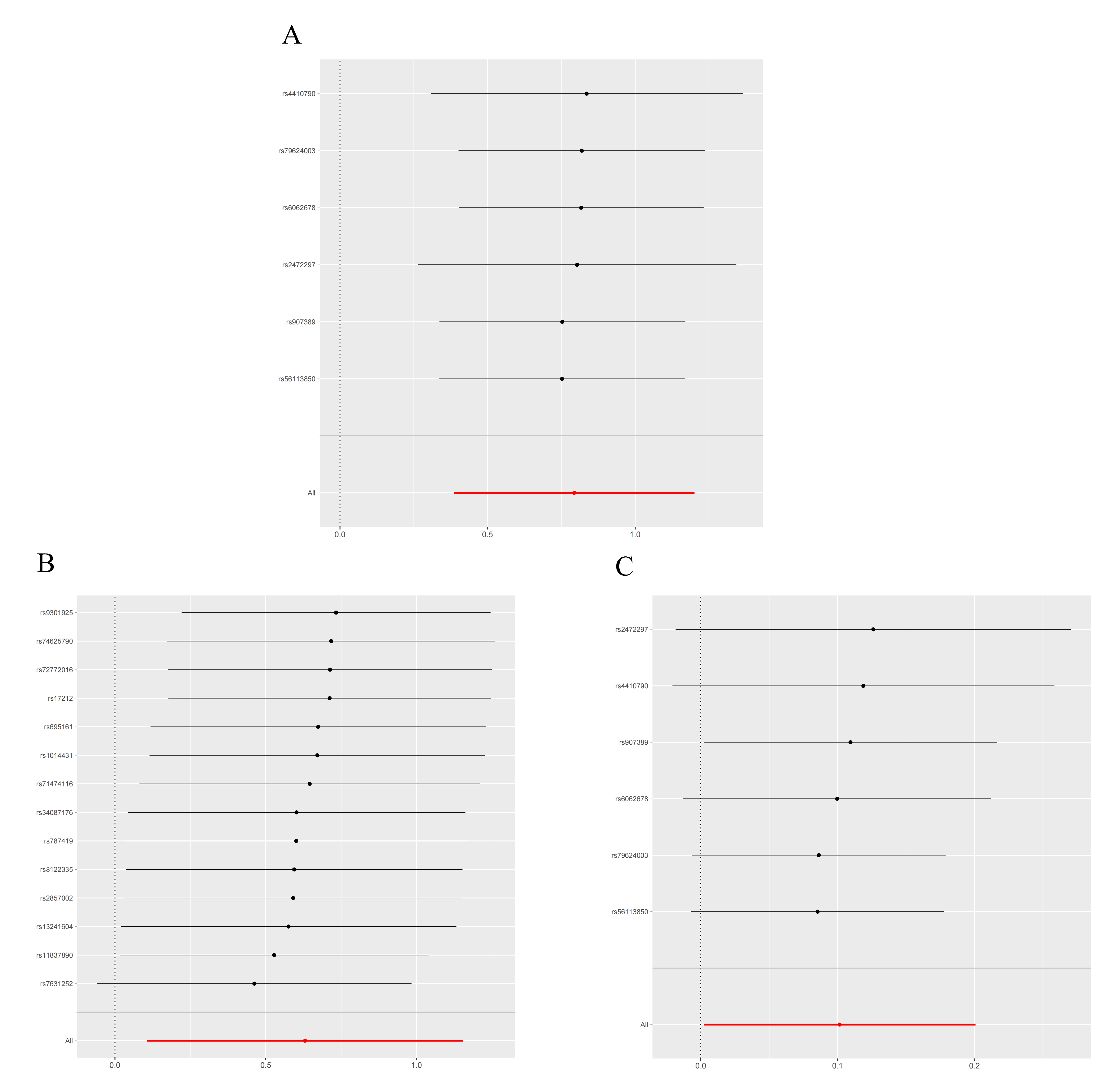
Figure S2. MR leave-one out sensitivity analysis for (A) coffee consumption on diabetic angiopathy (DA), (B) *Lawsonibacter sp002161175 abundance* on DA, and (C) coffee consumption on *Lawsonibacter sp002161175* abundance.
